# A Case of Dilated Cardiomyopathy Associated with 3-Hydroxy-3-Methylglutaryl-Coenzyme A (HMG CoA) Lyase Deficiency

**DOI:** 10.1155/2009/183125

**Published:** 2009-11-04

**Authors:** Alexander A. C. Leung, Alicia K. Chan, Justin A. Ezekowitz, Alexander K. C. Leung

**Affiliations:** ^1^Department of Medicine, University of Alberta Hospital, University of Alberta, Edmonton, AB, Canada T6G 2B7; ^2^Department of Medical Genetics, University of Alberta/Stollery Children's Hospital, University of Alberta, Edmonton, AB, Canada T6G 2B7; ^3^Department of Pediatrics, Alberta Children's Hospital, University of Calgary, Calgary, AB, Canada T2M 0H5

## Abstract

3-hydroxy-3-methylglutaryl-coenzyme A (HMG CoA) lyase deficiency is an inborn error of metabolism characterized by impairment of ketogenesis and leucine catabolism resulting in an organic acidopathy. In 1994, a case of dilated cardiomyopathy and fatal arrhythmia was reported in a 7-month-old infant. We report a case of dilated cardiomyopathy in association with HMG CoA lyase deficiency in a 23-year-old man with the acute presentation of heart failure. To our knowledge, this is the first case reported in an adult.

## 1. Introduction

Described in 1976, 3-hydroxy-3-methylglutaryl-coenzyme A (HMG CoA) lyase deficiency is a rare autosomal recessively inherited inborn error of metabolism characterized by impairment of ketogenesis and leucine catabolism resulting in attacks of metabolic acidosis, hypoglycemia (without ketosis), and a characteristic pattern of elevated urinary organic acids: 3-hydroxy-3-methylglutaric, 3-methylgutaconic, 3-methylglutaric, and 3-hydroxyisovaleric acids. Patients typically present within the first year of life [1]. This condition has been associated with hepatomegaly, pancreatitis, seizures, and hyperammonemia. In 1994, Gibson et al. reported a fatal case of dilated cardiomyopathy in a 7-month-old infant with HMG CoA lyase deficiency [2]. We report the first case of dilated cardiomyopathy in an adult with HMG CoA lyase deficiency.

## 2. Case Presentation

A 23-year-old man with HMG CoA lyase deficiency presented with two months of exertional dyspnea and cough with progressive malaise and lethargy. He denied any weight gain, swelling, orthopnea, or paroxysmal nocturnal dyspnea. He was diagnosed at age 9 months and is the only child of fourth cousin parents of Portugese ancestry. There is no family history of inherited metabolic disease, heart disease, or sudden unexplained deaths. He was initially treated with levo-carnitine, but had stopped taking it for 5 years as he reported unpleasant “fishy” body and urine odour.

Examination revealed a blood pressure of 115/87 mmHg, a regular heart rate of 122 beats per minute, respiratory rate 30 breaths per minute on room air. The jugular venous pulsation was elevated above the angle of the jaw. His apex was laterally displaced. A normal S1, physiologically split S2, and S3 were auscultated. The remainder of the cardiovascular, respiratory, abdominal, musculoskeletal, neurological, and dermatological examination was unremarkable.

Investigations revealed a normal complete blood count. Brain natriuretic peptide was elevated at 2067 pg/mL and troponin I levels were undetectable. Urinary organic acid analysis by mass spectrometry detected massive amounts of 3-hydroxy-3-methylglutaric and methylglutaconic acids. Serum total free carnitine was 11 *μ*mol/L (normal 16–66 *μ*mol/L), total carnitine 17 *μ*mol/L (normal 25–88 *μ*mol/L), and beta-hydroxybutyrate was undetectable. Creatinine kinase and thyroid stimulating hormone were normal. Viral studies were negative. Electrocardiogram showed sinus tachycardia. Chest radiograph showed an increased cardiothoracic ratio, a small left pleural effusion, and normal pulmonary vascularity and interstitial markings. Cardiac magnetic resonance (CMR) imaging revealed severe left ventricular enlargement (left ventricular end diastolic volume of 354 mL) with an ejection fraction of 15% and an apical thrombus, severe right ventricular dysfunction, severe biatrial enlargement, but no valvular disease. There was no evidence of myocarditis [3].

The diagnosis of heart failure secondary to dilated cardiomyopathy was made in association with HMG CoA lyase deficiency and carnitine deficiency. He was treated with carvedilol, ramipril, furosemide, spironolactone, and warfarin. A dietary treatment with moderate protein restriction, avoidance of high-fat, and increasing carbohydrate consumption to avoid fasting was reinforced. Levo-carnitine was restarted. After 10-months of follow-up, he had New York Heart Association (NYHA) class I symptoms, but no significant recovery of left ventricular function.

## 3. Discussion

It has been previously reported that a 7-month old boy with HMG CoA lyase deficiency manifested a fatal arrhythmia associated with a dilated cardiomyopathy [2]. Autopsy confirmed the presence of a dilated cardiomyopathy with vacuolar cytoplasmic changes in the absence of myocarditis, and negative viral serologies and cultures. In our patient, myocarditis was excluded using CMR [3, 4]. We postulate that his cardiomyopathy resulted from impaired ketogenesis, intracellular fatty acid accumulation, and secondary carnitine deficiency.

**Figure 1:**
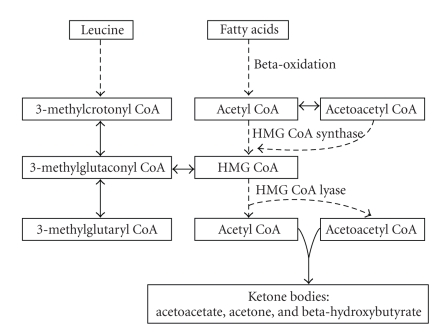
Biochemical pathways illustrating ketogenesis through the metabolism of fatty acids and leucine.

HMG CoA lyase catalyzes the last step in the catabolism of leucine and free fatty acids in ketogenesis (Figure 1). Under normal conditions, ketones are a minor energy substrate for the myocardium [5, 6]. More significant ketone utilization occurs during times of starvation, in heart failure, and with high-fat diets. In the setting of HMG CoA lyase deficiency, deprivation of this energy substrate may consequently contribute to myocardial dysfunction [5]. Ketone bodies, derived from beta-oxidation of free fatty acids, supply 60–90% of the total myocardial energy requirements, with the remaining energy needs met primarily by glucose [5]. Physiologically, fatty acid uptake is regulated by mitochondrial utilization, thereby preventing intracellular accumulation. In HMG CoA lyase deficiency, this regulative mechanism is impaired, and subsequent myocardial cytotoxicity and dysfunction result as a result of intracellular fatty acid accumulation [6].

Carnitine is essential for normal myocardial function [5, 7]. Fatty acids are transported from the cytosol into the mitochondria for beta-oxidation via the carnitine palmitoyltransferase shuttle system. In addition to its role in fatty acid transport, carnitine also serves as a free radical scavenger, and a cofactor in the oxidation of long-chain fatty acids, the metabolism of branched-chain amino acids, and nuclear transcription [5]. In HMG CoA lyase defiency, secondary carnitine deficiency results from esterification of free carnitine to 3-methylglutarylcarnitine. Levo-carnitine has been reported to attenuate left ventricular dilatation, and improve survival in patients with dilated cardiomyopathy and NYHA class III-IV symptoms [7]. Although our patient symptomatically improved with medical therapy and levo-carnitine supplementation, no recovery of left ventricular function was noted after 10 months of follow-up.

While the majority of patients with HMG CoA lyase deficiency present within the first year of life, it may rarely present during adulthood [1]. Adult survivors are felt to manifest a milder phenotype, and associated complications may therefore go unrecognized. Although a direct causal relationship between HMG CoA lyase deficiency and dilated cardiomyopathy has yet to be established, we provide a biochemical basis for our hypothesis: impaired ketogenesis, intracellular fatty acid accumulation, and secondary carnitine deficiency. To our knowledge, we report the first case of a dilated cardiomyopathy associated with HMG CoA lyase deficiency in adulthood. With report of this case, it is hoped that more confirmatory case reports would be forthcoming.

## Abbreviations

HMG CoA: 3-hydroxy-3-methylglutaryl-coenzyme A
CMR: Cardiac magnetic resonance

## References

[1] Reimão S, Morgado C, Almeida IT 3-Hydroxy-3-methylglutaryl-coenzyme A lyase deficiency: initial presentation in a young adult.

[2] Gibson KM, Cassidy SB, Seaver LH (1994). Fatal cardiomyopathy associated with 3-hydroxy-3-methylglutaryl-CoA lyase deficiency. *Journal of Inherited Metabolic Disease*.

[3] Hundley WG, Bluemke D, Bogaert JG (2009). Society for cardiovascular magnetic resonance guidelines for reporting cardiovascular magnetic resonance examinations. *Journal of Cardiovascular Magnetic Resonance*.

[4] Howlett JG, McKelvie RS, Arnold JMO (2009). Canadian cardiovascular society consensus conference guidelines on heart failure, update 2009: diagnosis and management of right-sided heart failure, myocarditis, device therapy and recent important clinical trials. *Canadian Journal of Cardiology*.

[5] Kodde IF, van der Stok J, Smolenski RT, de Jong JW (2007). Metabolic and genetic regulation of cardiac energy substrate preference. *Comparative Biochemistry and Physiology A*.

[6] Kayser MA (2006). Disorders of ketone production and utilization. *Molecular Genetics and Metabolism*.

[7] Allard ML, Jeejeebhoy KN, Sole MJ (2006). The management of conditioned nutritional requirements in heart failure. *Heart Failure Reviews*.

